# Platelet-Rich Fibrin Promotes Periodontal Regeneration and Enhances Alveolar Bone Augmentation

**DOI:** 10.1155/2013/638043

**Published:** 2013-03-26

**Authors:** Qi Li, Shuang Pan, Smit J. Dangaria, Gokul Gopinathan, Antonia Kolokythas, Shunli Chu, Yajun Geng, Yanmin Zhou, Xianghong Luan

**Affiliations:** ^1^UIC Brodie Laboratory for Craniofacial Genetics, 801 South Paulina, Chicago, IL 60612, USA; ^2^Department of Implantology, Stomatological Hospital, Jilin University, Changchun, Jilin 130021, China; ^3^Department of Endodontics, School of Dentistry, Harbin Medical University, Harbin, China; ^4^Department of Oral and Maxillofacial Surgery, UIC College of Dentistry, Chicago, IL, USA; ^5^The First Hospital of Jilin University, Jilin, China

## Abstract

In the present study we have determined the suitability of platelet-rich fibrin (PRF) as a complex scaffold for periodontal tissue regeneration. Replacing PRF with its major component fibrin increased mineralization in alveolar bone progenitors when compared to periodontal progenitors, suggesting that fibrin played a substantial role in PRF-induced osteogenic lineage differentiation. Moreover, there was a 3.6-fold increase in the early osteoblast transcription factor RUNX2 and a 3.1-fold reduction of the mineralization inhibitor MGP as a result of PRF application in alveolar bone progenitors, a trend not observed in periodontal progenitors. Subcutaneous implantation studies revealed that PRF readily integrated with surrounding tissues and was partially replaced with collagen fibers 2 weeks after implantation. Finally, clinical pilot studies in human patients documented an approximately 5 mm elevation of alveolar bone height in tandem with oral mucosal wound healing. Together, these studies suggest that PRF enhances osteogenic lineage differentiation of alveolar bone progenitors more than of periodontal progenitors by augmenting osteoblast differentiation, RUNX2 expression, and mineralized nodule formation via its principal component fibrin. They also document that PRF functions as a complex regenerative scaffold promoting both tissue-specific alveolar bone augmentation and surrounding periodontal soft tissue regeneration via progenitor-specific mechanisms.

## 1. Introduction

Regenerative oral medicine entails the replacement of tissues lost to disease or injury with physiologically equivalent engineered tissues. Often, tissues in the oral cavity are of complex nature with bordering mineralized and soft tissue components, both of which harbor unique progenitor populations residing within specialized extracellular matrix frameworks [1, 2]. Mimicking such complex environments by using chemically homogenous scaffolds and uniform stem cell populations is often challenging. Instead, recent approaches favor complex natural scaffolds that allow for repopulation with the patient's own cells, thereby producing an autologous tissue-engineered organ [3]. 

One such complex natural scaffold ideally suited for autologous tissue regeneration is platelet-rich fibrin (PRF), a second generation platelet concentrate developed as an improvement over the earlier introduced platelet-rich plasma (PRP) as an aid for tissue repair and regeneration [4]. In contrast to PRP, which was prepared by adding bovine thrombin and anticoagulants, PRF is generated from centrifuged blood and is strictly autologous. PRF predominantly consists of a fibrin matrix rich in platelet and leukocyte cytokines such as IL-1*β*, -4, and -6, and growth factors such as TGF-*β*1, PDGF-AB, and VEGF [5]. Fibrin gels exploit the final stage of the coagulation cascade in which fibrinogen molecules self-assemble into a highly biocompatible three-dimensional fiber network [6]. The combination of fibrins and cytokines within PRF becomes a powerful bioscaffold with an integrated reservoir of growth factors for tissue regeneration [7]. The suitability of PRF as a biologically active scaffold has been illustrated in a number of studies revealing proliferation and differentiation of osteoblasts and gingival fibroblasts [8, 9]. Clinical studies have demonstrated that PRF promotes soft tissue and bone regeneration [10, 11], as well as periodontal tissue regeneration [12, 13]. The ability of PRF to augment and regenerate compromised tissues may be enhanced in combination with bone substitutes such as Bio-Oss or autologous bone [14, 15]. Together, these studies have established PRF as a highly biocompatible and inductive scaffold useful for a broad range of tissue engineering applications. 

In the present study, we have hypothesized that PRF may provide a scaffold material for periodontal tissue regeneration. Earlier studies have reported the effect of PRF on periodontal cells [16, 17]. Here we have compared the effects of PRF on dental follicle, periodontal ligament, and alveolar bone cells [1] using microscopy as well as biological assays for migration, proliferation, mineralization, and gene expression. We also tested PRF scaffold properties in subcutaneous implants in nude mice. Finally, we report on the use of PRF as a scaffold for peri-implant alveolar bone augmentation in clinical use. Together, these studies for the first time characterize the tissue-specific biological effects of PRF as a bioactive scaffold to promote periodontal soft and hard tissue regeneration. 

## 2. Materials and Methods

### 2.1. Preparation of PRF

To prepare PRF, 10 mL of fresh blood from the precaval vein of a pig was collected into 10 mL glass-coated tubes without anticoagulants. All pigs used in this study were female with an average age of 3.1 months (range from 2.9 to 3.5 months). The platelet count of whole pig blood was 10^5^/*μ*L. This study was approved by the Ethics Committee of the University of Illinois at Chicago, USA. As described in previous studies [4, 18], samples were immediately centrifuged at 2100 rpm (approximately 400 g) for 12 minutes using a Beckman centrifuge. The PRF clots were concentrated between the red blood cell corpuscles at the bottom of the centrifuge tubes, and acellular plasma called platelet-poor plasma (PPP) at the top of the tubes. PPP was collected by pipetting the supernatant of the centrifuged blood preparation. After PPP removal, PRF clots were mechanically separated from red blood cells and gently compressed using gauze to drain the remaining fluid. 

Reproducibility of studies was ensured by maintaining consistent preparation conditions for PRF membranes, including centrifugation force and time, tube size, and platelet count of pig blood, and by completing blood collection procedures within 2 minutes. For *in vitro* studies, all cell culture wells were treated with one whole PRF clot consisting of identical amounts of fibrin, platelets, and white blood cells, and each PRF clot was freshly prepared from blood. 

### 2.2. Preparation of Conditioned Medium

To prepare conditioned medium, PRF membranes were soaked in 5 mL fresh DMEM medium without fetal bovine serum in 6-well cell culture plates. The conditioned medium was collected every 48 h, and fresh medium was added into wells after collection. 

### 2.3. Isolation of Human Dental Progenitor Cells

To generate human dental progenitor cells, healthy human teeth (patients ranging from 12 to 15 years) were extracted for orthodontic reasons in accordance with the human subjects protocol approved by UIC's Institutional Review Boards and the Office for the Protection Research Subjects. The dental follicle (DF) was dissected from developing tooth organs, and alveolar bone (AB) and periodontal ligament (PDL) were prepared from teeth with tooth roots already formed. Mesenchymal stem cells were isolated from the dental tissues after digestion with collagenase/dispase as described before [2]. 

### 2.4. MTT Cell Proliferation Assay

PDL, DF, and AB cells (10^4^ cells/well) were seeded into 96-well plates and cultured for 8 hours. After cells were attached, each well was washed twice with phosphate-buffered saline solution (PBS), and either PRF-conditioned medium + 10% FBS or DMEM + 10% PPP + 10% FBS was added to the well. DMEM + 10% FBS was used as a control. Prior to the termination of culture, cells were incubated in MTT solution (2 *μ*g/mL of MTT in DMEM with 2% FBS) for 4 hours. To quantify proliferative activity, the MTT-stained cells were lysed in HCL/isopropanol, and the absorbance was detected at 570 nm with background subtraction at 630 nm. Cell proliferation was detected on a daily basis over the entire 7-day culture period. 

### 2.5. Chemotaxis Assay

Cell migration assays were performed using a FluoroBlok-24-multiwell insert system (DB Biosciences, Bedford, MA). PRF-conditioned medium, DMEM + 10% PPP, or DMEM was added into separate wells of the lower chambers of the FluoroBlok system. Serum-starved cells (10^5^) were seeded into each insert and allowed to migrate to the bottom of the membrane for 12 hours. The nonmigrated cells on the upper surface of the membrane were removed with a cotton swab, and the migratory cells that were attached to the lower surface were stained with DAPI. The numbers of migrated cells per membrane were counted under a Leica DMRX fluorescent microscope. 

### 2.6. Induction of Osteogenic Differentiation, Alkaline Phosphatase (ALP) Activity Assessment, and Alizarin Red S Staining

PDL, DF, and AB were seeded into 6-well plates at a concentration of 10^4^ cells/well and cultured for 8 hours. After cell attachment, PRF membranes were added to individual wells, soaked, and subjected to osteogenic treatment, which included addition of osteogenic medium (OM), DMEM + 10% PPP + 10% FBS, or DMEM to each individual well. The following four groups were employed in this study: (i) PRF in DMEM with 10% FBS, (ii) 10% PPP in DMEM with 10% FBS, (iii) osteogenic medium with 10% FBS, and (iv) DMEM with 10% FBS. To test the effect of fibrin as the major PRF component on periodontal progenitor differentiation and mineralization, fibrin was used to coat otherwise untreated cell culture dishes and compared with vacuum gas plasma-treated and plasma-untreated dishes. To generate fibrin, equal amounts of fibrinogen (100 mg/mL) and thrombin (500 *μ*/mL) were mixed prior to application. Subsequently, treated cells were cultured for an additional 7, 14, or 21 days. For alkaline phosphatase activity assays, cells were washed and stained with NBT/BCIP (Roche Diagnosis GmbH, Mannheim, Germany) after rinsing with PBS. For quantification of mineralized nodules, cells were fixed with methanol and stained with 10% alizarin red solution. Mineralized nodules were identified as red spots on the culture dish. Alkaline phosphatase activity was determined after one week and alizarin red S staining was performed after three weeks. Wells were scanned and the integrated optical density (average intensity/object density) of the stained area was calculated using the Image Pro Plus 6.0 software (Mediacy, Chicago, IL, USA).

### 2.7. RNA Extraction and RT PCR

Total RNAs were isolated from cultured cells using the TRIZOL LS Reagent (Invitrogen, Carlsbad, CA, USA) according to the manufacturer's instructions. Two micrograms of total extracted RNA was applied toward cDNA generation with the Sprint RT Complete kit (Clontech, Mountain View, CA, USA). PCR primers were designed based on EMBL/GenBank searches, and primer sequences were as follows: *RUNX2: *5′-TTA CTT ACA CCC CGC CAG TC-3′ (sense), 3′-CAC TCT GGC TTT GGG AAG AG-5′ (antisense); *MGP*: 5′-CCC TCA GCA GAG ATG GAG AG-3′ (sense), 3′-GCTTCCCTATTGAGCTCGTG-5′ (antisense), **β*-ACTIN*: 5′-GCA TGG GTC AGA AGG ATT CCT-3 (sense), and 3′-TCG TCC CAG TTG GTG ACG AT-5′ (antisense). Real-time PCR was performed using sequence-specific SyberGreen primers and the ABI Prism 7000 sequence detection system (Applied Biosystems, Foster City, CA, USA). Reaction conditions were as follows: 2 min at 50°C (one cycle), 10 min at 95°C (one cycle), 15 sec at 95°C, and 1 min at 60°C (40 cycles). Samples were normalized using **β*-actin*. The analyses were performed in triplicate for three independent experiments to confirm reproducibility of the results. Relative expression levels were calculated using the 2^−ΔΔCt^ method [19], and values were graphed as the mean expression level ± standard deviation.

### 2.8. Protein Extraction and Western Blot Analysis

For western blot evaluation of the effect of PRF on RUNX2 in cultured periodontal progenitors, cells were collected, and equal amounts of protein extracts in a lysis buffer containing 100 mM Tris HCl pH 9.0, 200 mM KCl, 25 mM EGTA, 36 mM MgCl_2_, 2% deoxycholic acid, and 10% DTT v/v were subjected to SDS-polyacrylamide gel electrophoresis. The separated proteins were transferred to a PVDF membrane (Immobilon P, Millipore) and the membrane was incubated with anti-RUNX2 or anti-GAPDH primary antibodies (Abcam, Cambridge, MA, USA). Immune complexes were detected with peroxidase-conjugated secondary antibody (Molecular Probes, Carlsbad, CA, USA) and enhanced by chemiluminescence reagents (Pierce Biotechnology, Rockford, IL, USA). The amounts of protein expression were compared after normalization against GAPDH as an internal calibrator in each lane.

### 2.9. Subcutaneous Implant

Fresh PRF membranes measuring 5 mm^2^ in diameter and 1 mm^2^ in thickness were implanted under the cutis of nude mice (male, 6 weeks of age, Charles River). After 7 and 14 days of implantation, nude mice were sacrificed and implants were dissected for histological analyses.

### 2.10. Mallory Staining

Implants were fixed with 10% formalin at 4°C and processed for paraffin sections. Deparaffinized and rehydrated sections were stained in a series of toluidine blue, acid fuchsine, and aniline, for 2 minutes, respectively. 

### 2.11. Clinical Application

Two clinical pilot studies were conducted to test the effect of PRF on peri-implant sites in human patients. These studies were approved by the Ethics Committee of the Jilin University Health Science Center, Changchun, China. Informed consent was obtained from patients before surgery. Studies were conducted at Jilin University Stomatological Hospital and were consistent with the principles enunciated in the Declaration of Helsinki on the protection of human subjects. All patients selected for implant placement and PRF treatment were required to demonstrate superior oral hygiene with no or minimal plaque. Two Asian healthy adults, one male aged 28 years and one female aged 22 years, served as volunteers. Both patients demonstrated evidence of residual root tips prior to tooth extraction according to radiographs. For the rehabilitation of the patient, the fractured incisor (patient I) and the decayed molar (patient II) were extracted. Implant dimensions slightly varied between patients (13 mm × 3.5 mm, patient I, and 12 mm × 4.8 mm, patient II). Both patients exhibited substantial gaps between implant and alveolar bone socket. To close the gap between implant and extraction socket, PRF was prepared individually from each patient's blood sample, and squeezed PRF membranes were placed between implant and adjacent bone. Subsequently, the surgical site was closed using silk sutures. As a followup, radiographs were taken immediately and three months after surgery. Wound healing and bone levels were examined using intraoral photographs and oral radiographs. To calculate bone levels, implant dimensions served as a reference. 

### 2.12. Statistical Analysis

Statistical analysis was performed using the SPSS 13.0 software (Chicago, IL, USA) and Student's *t*-test. Data were expressed as mean ± SD. For all tests, statistical significance was assigned when *P* < 0.05. Experiments were repeated 3 times to ensure reproducibility.

## 3. Results

### 3.1. PRF Clot Prepared from Fresh Blood Contained Fibrin, White Blood Cells, and Leukocytes

**Figure 1:**
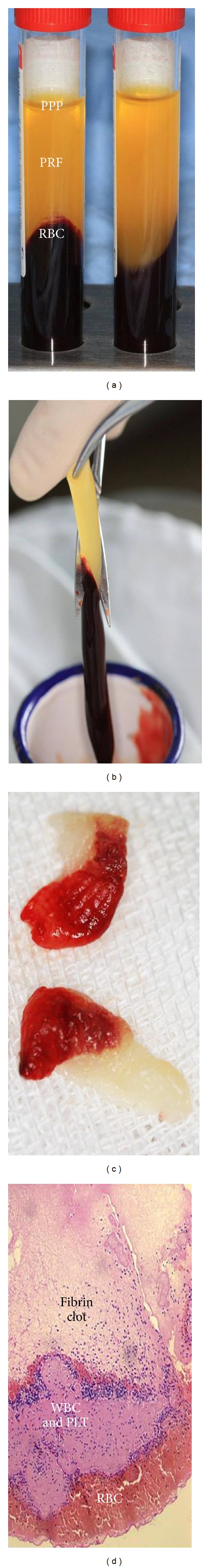
Preparation and structure of platelet-rich fibrin (PRF). (a) shows three fractions of whole blood generated by low-speed centrifugation: platelet-poor plasma (PPP), platelet-rich fibrin (PRF), and red blood cells (RBC). At this stage, PRF and RBC have formed a gel, while the PPP fraction remains liquid. (b) Mechanical separation of the RBC fraction from the PRF fraction after decanting the PPP fraction. (c) Generation of solid PRF after liquid removal. (d) 5 *μ*m paraffin section through solidified PRF generated in (c). The section contains three portions: (i) the cell-free fibrin clot, (ii) the buffy coat portion containing white blood cells (WBC) and platelets (PLT), and (iii) the red blood cell (RBC) portion. Note that the majority of the white blood cells and platelets are trapped in the buffy coat.

The focus of the present study was to determine the suitability of PRF for biological reengineering of complex periodontal tissues, including soft and mineralized tissues. As a first step, we have analyzed the histological composition of the PRF scaffold used in our studies. For our experiments, PRF was generated by low-speed centrifugation from whole blood. Centrifugation resulted in separation of three distinct fractions: platelet-poor plasma (PPP), platelet-rich fibrin (PRF), and red blood cells (RBC) (Figure 1(a)). The PRF fraction was further isolated by decanting the soluble PPP fraction and by mechanically removing the RBC fraction (Figure 1(b)). Liquid removal from the PRF fraction through mechanical pressure between gauze layers resulted in a fairly solid, gel-like material (Figure 1(c)). H&E-stained paraffin sections through the PRF clot revealed three portions: (i) the cell-free fibrin clot, (ii) the buffy coat portion containing white blood cells (WBC) and platelets (PLT), and (iii) the red blood cell (RBC) portion (Figure 1(d)). For the experiments described herein, the entire PRF clot was used, including fibrin, white blood cells, and platelets. The red blood cells immediately attached to the PRF clot were not further separated. 

### 3.2. PRF Significantly Increased Periodontal Progenitor Cell Migration and Proliferation *In Vitro* When Compared to PPP and DMEM Media

**Figure 2:**
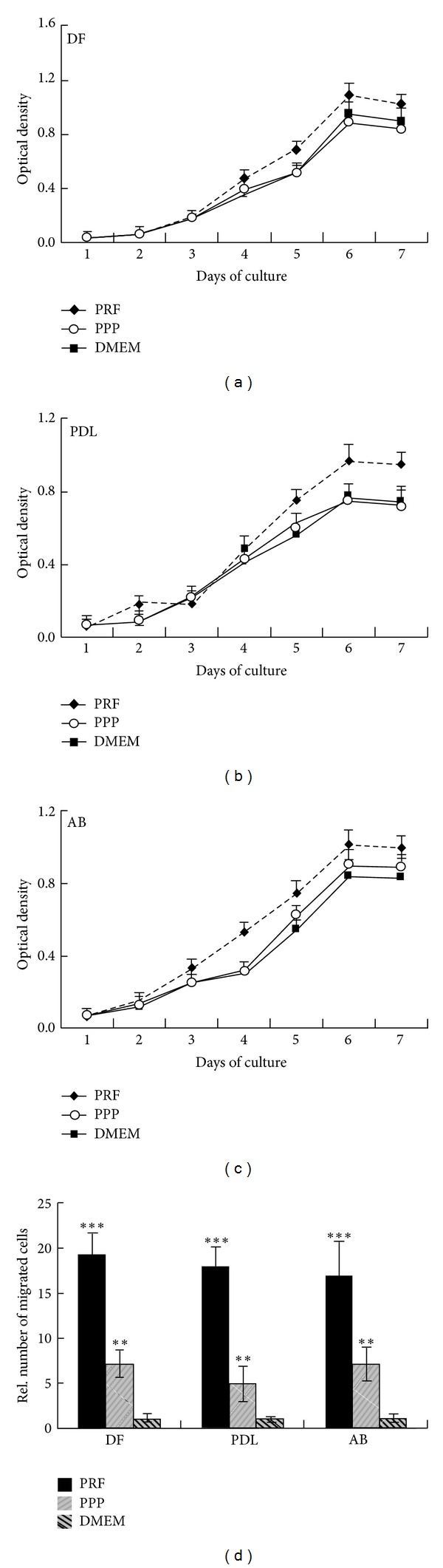
Effects of PRF when compared to PPP and medium alone on proliferation and migration of periodontal progenitor populations. Figures 2(a)–2(c) illustrate the results of MTT proliferation assays when dental follicle progenitors (DF, (a)), periodontal ligament progenitors (PDL, (b)), and alveolar bone osteoblast progenitors (AB, (c)) were cultured on PRF-related substrates. The three different substrates used in this proliferation study, PRF, PPP, and DMEM media, are distinguished by line patterns (Figure 2(a)). (d) Difference in chemotaxis behavior between periodontal progenitors when cultured in PRF-conditioned media, PPP, and DMEM medium. Level of significance was calculated in comparison to the DMEM-treated cells within each group. **P < 0.01 and ***P < 0.001.

Effective biocompatible tissue engineering scaffolds provide a template for cells to migrate and proliferate [20]. To determine the ability of PRF-based scaffolds to promote cell proliferation and migration, periodontal progenitors were cultured for up to 7 days using PRF, PPP, or DMEM alone (Figure 2). PRF was compared with PPP to compare the effects of a fibrin-rich (PRF) and a fibrin-poor (PPP) blood centrifugate on periodontal cells. Our cell proliferation assays using PDL fibroblasts, dental follicle progenitors, and alveolar bone osteoblasts demonstrated a gradual increase in cell density over a culture period of 7 days with all three culture conditions (Figures 2(a)–2(c)). PRF resulted in higher proliferation rates than DMEM medium alone or platelet-poor plasma (PPP) (Figures 2(a)–2(c)). After 7 days of culture, the PRF-induced increase in proliferation significantly surpassed the PPP- and DMEM-induced proliferation rate in all cell cultures examined (30.1% for DF, 34% for PDL, and 22.4% for AB; *P* < 0.05). Migration assays revealed that PRF caused a highly significant (*P* < 0.0001) and higher than 15-fold increase (19.3-fold for DF, 18-fold for PDL, and 17.1-fold for AB) in the number of migrated cells when compared to DMEM medium, while platelet-poor plasma (PPP) only caused a moderate 5–10-fold increase in the number of migrated cells (7.8-fold for DF, 5-fold for PDL, and 7.1-fold for AB).

### 3.3. PRF Almost Equaled Osteogenic Medium in Its Effect on Mineralization Behavior of Cultured Periodontal Progenitors and in Comparison to DMEM and PPP

**Figure 3:**
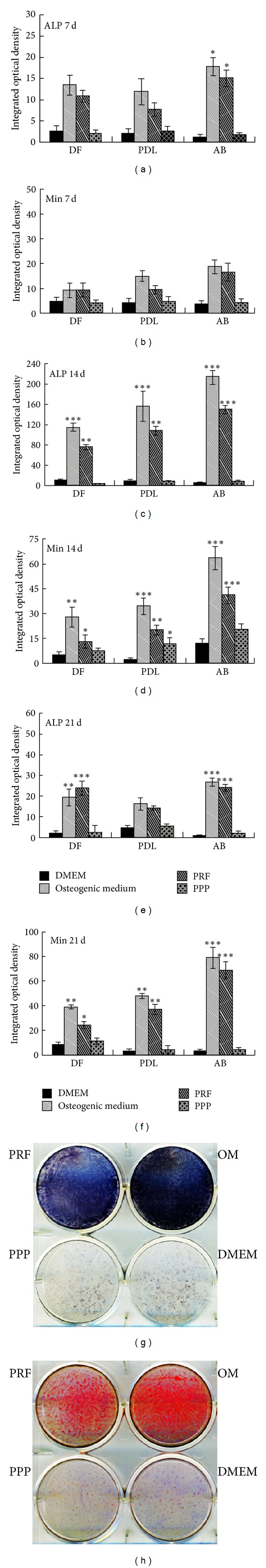
Effect of PRF, PPP, and DMEM media on mineralization behavior of periodontal progenitor populations. (a), (c), (e), and (g) are alkaline phosphatase staining assays and (b), (d), (f), and (h) are alizarin red S mineralization assays. In (a)–(f), alkaline phosphatase ((a), (c), and (e)) or alizarin red ((b), (d), and (f)) staining in periodontal progenitor cells cultured for 7, 14, and 21 days were compared. Different coculture conditions (PRF, PPP, OM, and DMEM) are distinguished by different bar patterns which are identified in the bar legend above (b). The three periodontal progenitor populations compared in this study, dental follicle (DF), periodontal ligament (PDL), and alveolar bone (AB) are labeled on the *x*-axis of the graphs in (a)–(f). (g) and (h) illustrate the differences in mineralization behavior between alveolar bone progenitors cocultured with PRF, OM, PPP, and DMEM for 14 days; (g) is a photograph of the alkaline phosphate-stained 6-well plate and (h) is a photograph of the alizarin red-stained 6-well plate. Level of significance was calculated in comparison to the DMEM-treated cells within each group. *P < 0.05, **P < 0.01, and ***P < 0.001.

Previous studies have established the osteogenic potential of periodontal progenitors, including dental follicle and periodontal ligament progenitors [21, 22]. However, periodontal progenitors do not form bone or other mineralized tissues in routine tissue engineering applications without osteoinduction [2]. Moreover, *in vivo* studies have demonstrated the suitability of fibrin-based scaffolds for bone tissue engineering [23]. We therefore asked the question to what extent PRF unleashes the osteogenic potential of periodontal progenitors. In our studies, DF, PDL, and AB cells were subjected to coculture with platelet-rich fibrin (PRF), osteogenic medium (OM), platelet-poor plasma (PPP), or Dulbecco's modified Eagle's medium (DMEM), and alkaline phosphatase and alizarin red staining were used to evaluate osteoblast activity and mineralized nodule formation. After 7 days, alkaline phosphatase levels as a result of PRF coculture increased 4.1-fold (DF), 3.7-fold (PDL), and 12.6-fold (AB), while OM coculture resulted in an 5.1-fold (DF), 5.8-fold (PDL), and 14.8-fold (AB) increase of alkaline phosphatase levels (Figure 3(a)). These effects were highly significant (*P* < 0.01), while there was no significant difference in alkaline phosphatase levels between PPP-treated cells and DMEM-treated cells. After 14 days, alkaline phosphatase levels as a result of PRF coculture increased 7-fold (DF), 10.8-fold (PDL), and 25-fold (AB), while OM coculture resulted in a 10.6-fold (DF), 15.7-fold (PDL), and 35.8-fold (AB) increase of alkaline phosphatase levels (Figure 3(c)). Differences once more were highly significant (*P* < 0.001 for DF/OM, *P* < 0.01 for DF/PRF, *P* < 0.001 for PDL, and *P* < 0.0001 for AB), while alkaline phosphate levels of PPP-treated and DMEM-treated cells were at the same level. Alkaline phosphatase levels after 21 days of culture were decreased compared to 14-day levels, but still are higher than those after 7 days. Specifically, alkaline phosphatase levels in the PRF-treated group increased 10.4-fold (DF), 2.9-fold (PDL), and 20.1-fold (AB), while alkaline phosphatase levels in the OM-treated group increased 8.5-fold (DF), 3.3-fold (PDL), and 22.3-fold (AB), once more at statistically highly significant levels (*P* < 0.01 for DF and PDL, and *P* < 0.001 for AB) (Figure 3(e)). 

In addition to alkaline phosphate levels, alizarin red S staining was performed as a means to assess matrix mineralization. After 7 days, alizarin red S staining as a result of PRF coculture increased 2-fold (DF), 2.3-fold (PDL), and 4.9-fold (AB), while OM coculture resulted in an increase of alizarin red S staining by 1.9-fold (DF), 3.7-fold (PDL), and 5.5-fold (AB) (Figure 3(b)). These effects were highly significant (*P* < 0.05 for DF, *P* < 0.01 for PDL/OM, *P* < 0.05 for PDL/PRF, and *P* < 0.01 for AB), while there was no significant difference in alizarin red S staining between PPP-treated cells and DMEM-treated cells. After 14 days, alizarin red S staining as a result of PRF coculture increased 2.6-fold (DF), 9.3-fold (PDL), and 3.3-fold (AB), while OM coculture resulted in an increase of alizarin red S staining by 5.6-fold (DF), 15.9-fold (PDL), and 5.2-fold (AB), when compared to DMEM-treated cells (Figure 3(d)). Differences once more were highly significant (*P* < 0.01 for DF/OM, *P* < 0.05 for DF/PRF, *P* < 0.001 for PDL/OM, *P* < 0.01 for PDL/PRF, *P* < 0.0001 for AB/OM, and *P* < 0.001 for AB/PRF). Alizarin red S staining after 21 days of culture was further enhanced, with staining levels in the PRF-treated group increased to 2.8-fold (DF), 10.8-fold (PDL), and 20.2-fold (AB), while alizarin red S staining in the OM-treated group increased 4.5-fold (DF), 14-fold (PDL), and 23.1-fold (AB), once more at statistically highly significant levels (*P* < 0.01 for DF, *P* < 0.001 for PDL, and *P* < 0.0001 for AB) (Figure 3(f)). Effects of PRF, OM, PPP, and DMEM on alkaline phosphatase levels (Figure 3(g)) and alizarin red S staining (Figure 3(h)) as mineralization indicators after 14 days of alveolar bone progenitor culture were documented. 

### 3.4. PRF Significantly Enhanced RUNX2 Expression and Reduced MGP Expression in AB Progenitors, While It Affected DF and PDL Cells to a Lesser Degree

**Figure 4:**
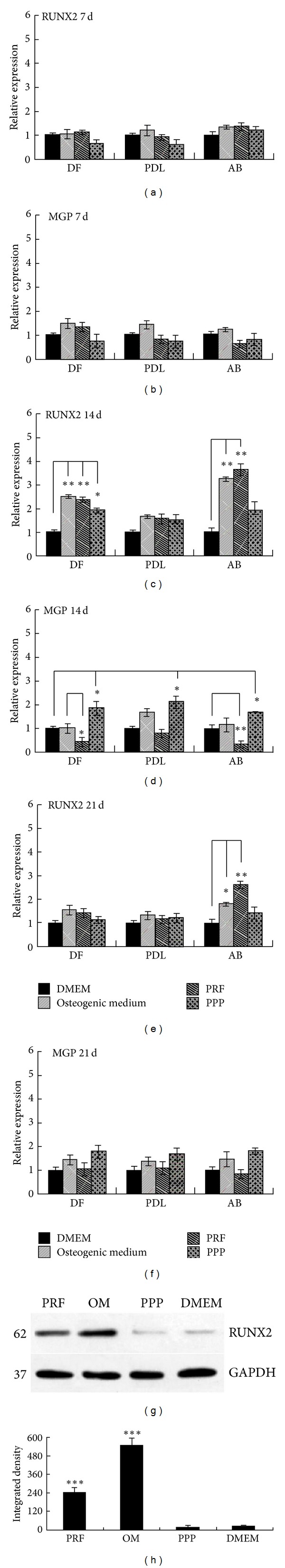
Differences in mineralization-associated gene expression patterns in cocultures of periodontal progenitors and centrifuged blood derivatives. (a), (c), and (e) are real-time RT-PCR assays for the osteoblast transcription factor Runx2, and (b), (c), and (f) are real-time RT-PCR assays for the calcification inhibitor Matrix Gla Protein (MGP). Different coculture conditions (PRF, PPP, and DMEM) are distinguished by different bar patterns identified in the upper right corner of the figure. The three periodontal progenitor populations compared in this study, dental follicle (DF), periodontal ligament (PDL), and alveolar bone (AB), are labeled on the *x*-axis of the graphs in (a)–(f). (g) is a western blot comparing Runx2 protein levels generated by AB cells cocultured either with PRF, OM, PPP, or DMEM. GAPDH served as a control. (h) is the corresponding densitometry evaluation. Level of significance was calculated in comparison to the DMEM-treated cells within each group. *P < 0.05, **P < 0.01, and ***P < 0.001.

To test whether changes in mineralization behavior of periodontal progenitors as a result of PRF application or treatment with osteogenic medium (OM) corresponded to the changes in gene expression levels, expression levels of two osteogenic modulators, the osteoblastic differentiation transcription factor *RUNX2* [24] and the extracellular matrix mineralization inhibitor [25] matrix GLA protein (*MGP*). After 7 days of culture, neither *RUNX2* nor *MGP* was substantially affected by PRF or OM in any of the three cell types investigated (Figures 4(a) and 4(b)). After 14 days of culture, *RUNX2* expression in all three cell types was significantly increased as a result of PRF, OM, and PPP application (Figure 4(c)). Notably, PRF and OM treatment had a remarkable effect on *RUNX2* levels in AB cells when compared to DF and PDL cells. While *RUNX2* expression in DF and PDL progenitors approximately doubled in response to OM and PRF exposure, *RUNX2* levels increased 3.3-fold after OM application and 3.6-fold after PRF application in AB cells (Figure 4(c), *P* < 0.01). This general trend was retained after 21 days, albeit at slightly reduced levels: OM caused a 1.8-fold increase in *RUNX2* expression in AB cells and PRF resulted in a 2.6-fold increase in *RUNX2* expression in AB cells, while there was little effect in the other two progenitor cell populations (Figure 4(e)). On western blots, PRF resulted in a 10.4-fold increase and OM in a 23.52-fold increase in *RUNX2* levels in AB cells cultured for 2 weeks when compared to the DMEM group (Figures 4(g) and 4(h), *P* < 0.001). As mentioned above, *MGP* was little affected by PRF or OM in any of the cell types after one week of culture (Figure 4(b)). Two or three weeks in the presence of PPP resulted in an approximately 2-fold elevation of *MGP* in all three cell types (Figures 4(d) and 4(f), *P* < 0.05). Most notable was a 3.1-fold reduction in *MGP* expression as a result of PRF application in AB cells after 2 weeks (*P* < 0.01) and a 2-fold PRF-induced reduction in *MGP* expression in DF cells (Figures 4(d) and 4(f), *P* < 0.05). There was still a 1.2-fold reduction in *MGP* expression in AB progenitors as a result of PRF treatment after 3 weeks of culture, while there were few differences in all other groups and cells after three weeks of culture, with the exception of the *MGP* upregulation caused by PPP (Figure 4(f)). 

### 3.5. Fibrin-Enhanced Alkaline Phosphatase Activity and Mineralization in AB Progenitors

**Figure 5:**
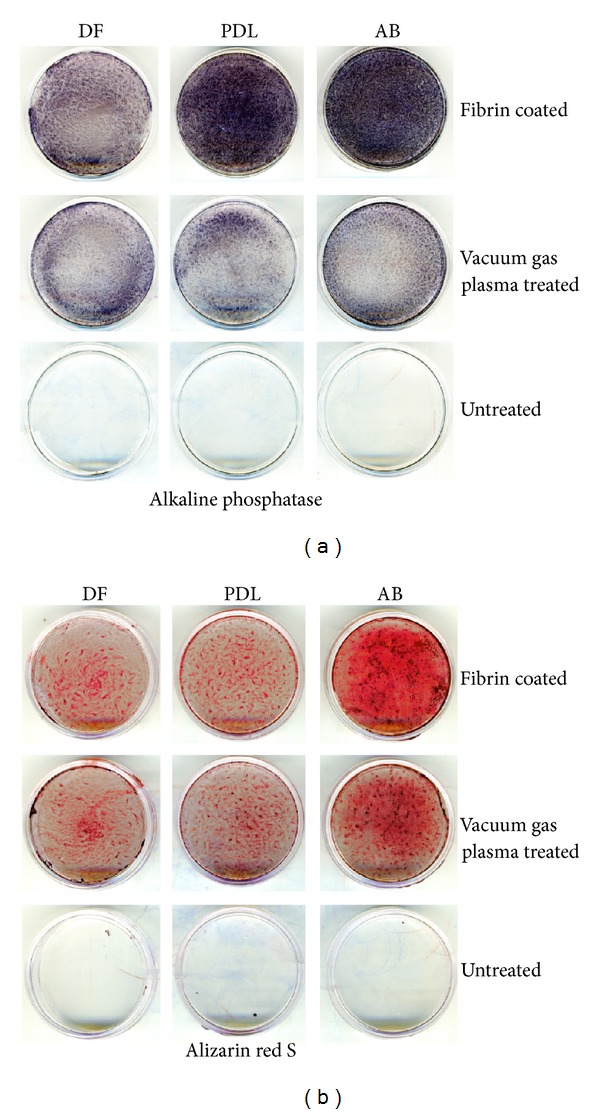
Effect of the major PRF component, fibrin, on periodontal progenitor attachment and mineralization. Osteogenic lineage differentiation (a) and mineralized nodule formation (b) of dental follicle cells (DF), periodontal progenitors (PDL), and alveolar bone progenitors (AB) on fibrin-coated dishes, vacuum gas plasma-treated tissue culture dishes, and untreated Petri dishes as revealed by alkaline phosphatase (a) and alizarin red S (b). Alkaline phosphatase activity was determined after one week and alizarin red S staining was performed after three weeks.

Studies so far indicated that PRF affects mineralization of periodontal progenitors, especially AB progenitors. To determine whether fibrin as the major structural component of the PRF scaffold affects mineralization behavior of periodontal progenitors, cells were cultured on fibrin-coated culture dishes, vacuum gas plasma-treated tissue culture dishes, and untreated Petri dishes, for 5 days, and stained for alkaline phosphatase activity (Figure 5(a)) or alizarin red S (Figure 5(b)). Alkaline phosphatase levels of PDL and AB progenitors were significantly elevated on fibrin-coated dishes when compared to vacuum gas plasma-coated dishes (4.57-fold for AB and 3.9-fold for PDL), while there was only a 1.77 elevation of DF cell progenitor alkaline phosphatase levels on fibrin-coated dishes. In contrast, alizarin red S as an indicator of mineralization was significantly increased in the AB progenitor group when compared to the PDL (4.76-fold increase when comparing AB versus PDL) and the DF groups (3.26-fold increase when comparing AB versus DF); AB culture alizarin red S levels were 2.15-fold higher on fibrin-coated dishes than on commercially pretreated dishes. 

### 3.6. PRF Scaffolds Integrated with Surrounding Tissues and Were Partially Replaced with Collagen Fibers Two Weeks after Subcutaneous Implantation in Nude Mice

**Figure 6:**
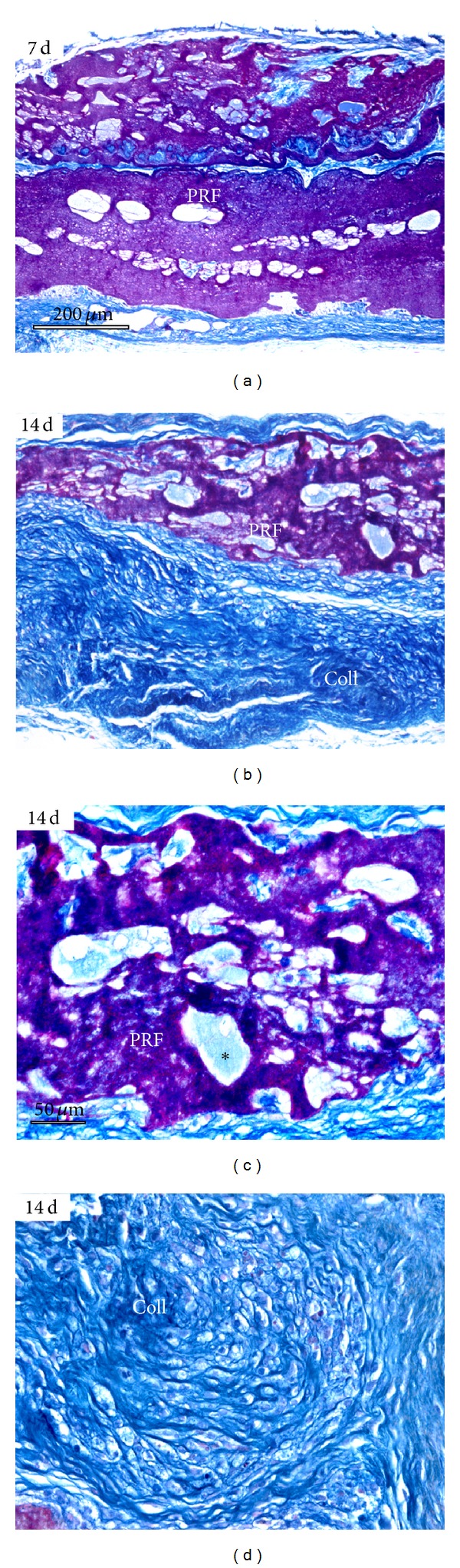
Evaluation of PRF as a scaffold in nude mice subcutaneous implants. This figure contains micrographs of paraffin sections through the center of the implant stained using Mallory's connective tissue stain. (a) After 7 days of implantation, the PRF implant remained fairly intact and was only surrounded by collagen fibers in the periphery of the implant. (b) After 14 days of implantation, the size of the PRF scaffold (PRF) was reduced and mostly replaced with collagen fibers (Coll, here stained in blue). (c) and (d) illustrate histology of the 14-day implant at higher magnification. Note small collagen fibers (light blue) present within the pores of the implant (∗, (c)). (d) demonstrates new collagen fiber formation (Coll) replacing the PRF implant. (a) and (b) bar = 200 *μ*m; (c) and (d) bar = 50 *μ*m.

To determine the suitability of PRF as a scaffold for tissue regeneration, PRF membranes were subcutaneously implanted in nude mice. One week after implantation, PRF membranes were completely surrounded by subcutaneous collagen fibers (Figure 6(a)). After 14 days, the thickness of the membrane was reduced to 0.5 mm and the remaining tissue had been replaced by dense collagen fibers (Figures 6(b) and 6(d)). At this time point, pores inside of the PRF membrane contained meshworks of thin collagen fibrils (Figure 6(c)). 

### 3.7. Soft Tissue Healing and New Bone Formation after PRF Application for Peri-Implant Periodontal Regeneration

**Figure 7:**
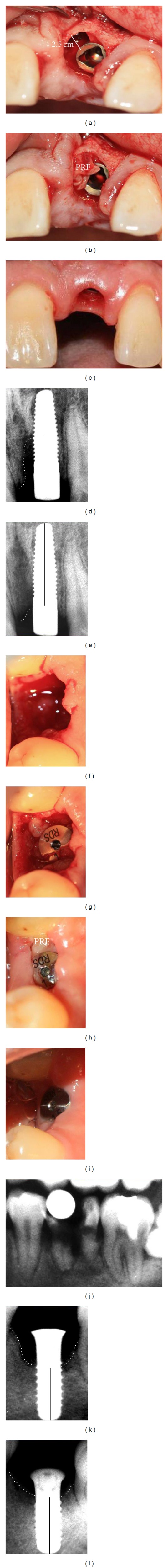
PRF application simultaneous with dental implant surgery. (a)–(e) Case I and (f)–(l) Case II, (a)–(c) and (f)–(i) are intraoral micrographs, and (d), (e), and (j)–(l) are X-rays. Case I: (a) reveals a 2.5 mm gap at the mesial aspect of the implant replacing the upper left incisor, (b) illustrates PRF placement in the gap between implant and adjacent alveolar bone, (c) demonstrates healing of the implant site and healthy gingiva three months after implant placement, (d) X-ray documenting gap between implant and alveolar bone immediately after implant placement (Case I), and (e) X-ray demonstrating new alveolar bone formation at the site of PRF application three months after implant placement (Case I). Case II: (f) extraction socket of a lower left molar immediately after tooth extraction, (g) implant placement and filling of bone-deficient peri-implant space with PRF, (h) healing of surgery site and PRF implant five days after surgery, (i) healthy soft tissue surrounding implant three months after implant placement, (j) X-ray of decayed lower left molar treated in Case II prior to extraction, (k) X-ray of implant taken immediately after surgery. (l) X-ray illustrating new alveolar bone formation three months after surgery. Note the disappearance of the bony defect between implant and adjacent sockets. The black bar drawn in the implant center on the radiographs ((d), (e), (k), and (l)) represents the distance from the foramen apical to the cervical-most margin of the surrounding alveolar bone.

To test the effect of PRF on peri-implant sites in human patients, clinical pilot studies were conducted. In these studies, PRF was placed into peri-implant tissue gaps of both human molars and incisors (Figure 7). Intraoral images illustrate soft tissue healing 5 days after surgery and PRF membrane placement (Figure 7(h)) as well as complete integration three months after surgery (Figures 7(c) and 7(i)). Radiographs demonstrate substantial new alveolar bone formation surrounding both implants (Figures 7(e) and 7(l) versus Figures 7(d) and 7(k)). On the mesial aspect of the incisor implant, there was a 5.4 mm alveolar bone gain (Figure 7(e) versus Figure 7(d)), while there was bone gain on both aspects of the molar implant amounting to approximately 4.9 mm (Figure 7(l) versus Figure 7(k)). Newly formed alveolar bone was radioopaque and contained trabeculae (Figures 7(e) and 7(l)). 

## 4. Discussion

In the present study, a series of experiments was conducted to determine the usefulness of PRF as a bioactive scaffold for periodontal tissue regeneration. In a first set of experiments, the effect of PRF on proliferation and migration of DF, PDL, and AB progenitors was investigated. DF, PDL, and AB progenitors were chosen as metabolically active periodontal progenitor populations [1]. In a second set of studies, the effect of PRF on periodontal progenitor mineralization was determined *in vitro*. In these experiments, classic osteoblast differentiation and mineralization markers such as alkaline phosphatase and alizarin red S as well as *RUNX2* expression were employed to gain detailed insight into the effect of PRF on mineralization behavior of periodontal progenitors. To ask whether the major PRF component fibrin was associated with the effect of PRF on periodontal progenitor mineralization, our alkaline phosphatase and alizarin red S assays were conducted on fibrin-coated dishes. Finally, subcutaneous implantation studies and human pilot studies were performed to determine the applicability of PRF as a scaffold for periodontal tissue regeneration. Together these studies demonstrated that the application of PRF in periodontal regeneration had two major benefits: (i) the promotion of soft tissue healing as explained by the effect of PRF on progenitor proliferation and migration and (ii) the induction of new alveolar bone formation as possibly facilitated by the fibrin-mediated effect of PRF on *RUNX2* expression, osteoblast differentiation, and matrix mineralization, and also by the alkaline phosphatase activity-stimulating effect of fibrin. 

 The PRF membranes used for the present study contained the entire PRF clot centrifuged from fresh blood, including fibrin, leukocytes, and platelets. By the very nature of this preparation, the PRF membranes used for the current study were three-component scaffolds that were not further biochemically dissected for individual components as the biological and therapeutic effects of the PRF scaffold have been reported to depend on the fresh preparation of an autologous blood fraction [4]. Moreover, the compound PRF membrane contains not only the structural scaffold components fibrin, leukocytes, and platelets, but also a multitude of growth factors such as TGF-*β*1, VEGF, IGFs, and PDGF-AB, as well as matricellular proteins such as thrombospondin-1 [26]. In the present study, the composition of the PRF membrane was kept as homogeneous as possible by maintaining strict and reproducible preparation conditions. We expect some limited variability among PRF samples due to differences between blood of individual host organisms, including humans. Nevertheless, our studies reported excellent reproducibility between individual PRF preparations from different donor individuals in terms of mineralization, proliferation, and migration effects, indicating that for the purpose of the present study, our PRF membranes elicited highly repeatable biological effects. 

 Our data demonstrated that PRF significantly improved periodontal progenitor cell proliferation and migration *in vitro* when compared to PPP and DMEM media. The effect of PRF on cell proliferation is well established and has been described in a wide variety of cell types, including periodontal ligament cells, osteoblasts, gingival fibroblasts, oral epithelial cells, BMSCs, preadipocytes, and prekeratinocytes [9, 27]. PRF's role in the stimulation of cell proliferation may be due to a gradual release of growth factors [8], some of which might be released by platelets and others might have been trapped within the fibrin scaffold and gradually diffused into the culture medium. Even though both PRF and PPP media are blood plasma preparations, PRF had a significantly stronger effect on proliferation than PPP. We attribute this effect to different subsets of cytokines in PRF and PPP and to the controlled release of cytokines trapped in PRF fibrin meshes [28]. Interestingly, there was a stronger effect of PRF on AB and DF cells than on PDL progenitors, and the PRF-induced elevation of proliferation occurred earlier in AB progenitors than in the other two cell types investigated, suggesting that the effects of PRF are tissue specific and favor AB osteoblasts over the other two cell types studied here. While the PRF-induced enhancement of cell proliferation remained in the 30% range over PPP or DMEM controls, the effect of PRF on cell migration was much more pronounced, featuring a 2.5-fold elevation compared to PPP and an approximately 20-fold elevation when compared to DMEM. This dramatic PRF-induced increase in cell migration has not been reported previously and might be explained by chemokines released by leukocytes trapped in PRF [29] or by the effect of soluble fibrin components on cell migration [30]. 

 From a clinical perspective, the reported effects of PRF on bone regeneration [31, 32] are of great interest for orthopedic applications because of the limitations of current strategies to enhance bone regeneration [33]. Moreover, alveolar ridge augmentation is of great benefit to implant dentistry because of the lack of supporting bone for implant placement [34]. Our *in vitro* studies indicated that PRF almost equaled osteogenic medium in its effect on mineralization behavior of cultured periodontal progenitors, surpassing DMEM and PPP by a significant margin. While the effect of PRF on osteoblast alkaline phosphatase levels [8] and on the formation of mineral nodules [35] has already been reported, we have demonstrated here that PRF significantly enhances the expression of the osteoblast differentiation transcription factor *RUNX2* and reduces expression of the mineralization inhibitor *MGP*, preferentially in alveolar bone osteoblast progenitors, and less so in dental follicle cells and periodontal ligament progenitors. We propose that this tissue-specific enhancement of osteoblast mineralization might be due to the enhanced alkaline phosphatase activity induced by the fibrin component of PRF (shown in the present study) and to the greater susceptibility of osteoblast cells to differentiate along osteogenic lineage pathways. 

 Both our *in vitro* and our clinical studies indicate that the benefit of PRF for bone regenerative procedures lies in its combined competency as a cell proliferation, migration, and wound-healing agent together with its tissue-specific ability to promote osteoblast differentiation and new bone formation. Our clinical pilot experiments revealed peri-implant bone gain of approximately 5 mm in conjunction with soft tissue healing around the implant site—a highly desirable outcome, in which we attribute the combined biological effects of PRF on gingival fibroblast and periodontal ligament cell migration, as well as alveolar bone osteoblast proliferation and mineralization. Moreover, PRF is a biodegradable scaffold as our studies have demonstrated that PRF subcutaneous implants were readily replaced with dense collagen even after 2 weeks of implant placement in nude mice, suggesting excellent biodegradability. The combinatorial effects of PRF on soft tissue healing and bone regeneration may limit its use in regenerative medicine applications in which calcification is not a desired outcome; however, our studies indicate that PRF contains a number of attributes ideally suited for its use as a scaffold for alveolar ridge augmentation and bone healing.
