# Erratum to “Platelet-Rich Fibrin Promotes Periodontal Regeneration and Enhances Alveolar Bone Augmentation”

**DOI:** 10.1155/2020/1794206

**Published:** 2020-02-26

**Authors:** Qi Li, Shuang Pan, Smit J. Dangaria, Gokul Gopinathan, Antonia Kolokythas, Shunli Chu, Yajun Geng, Yanmin Zhou, Xianghong Luan

**Affiliations:** ^1^UIC Brodie Laboratory for Craniofacial Genetics, 801 South Paulina, Chicago, IL 60612, USA; ^2^Department of Implantology, Stomatological Hospital, Jilin University, Changchun, Jilin 130021, China; ^3^Department of Endodontics, School of Dentistry, Harbin Medical University, Harbin, China; ^4^Department of Oral and Maxillofacial Surgery, UIC College of Dentistry, Chicago, IL, USA; ^5^The First Hospital of Jilin University, Jilin, China

In the article titled “Platelet-Rich Fibrin Promotes Periodontal Regeneration and Enhances Alveolar Bone Augmentation” [1], in Figure 1(a), the distance between the implant margin and the alveolar socket was incorrectly referred to as 2.5 cm. It should be corrected to 2.5 mm as mentioned in figure's description. The corrected figure is shown below.

## Figures and Tables

**Figure 1:**
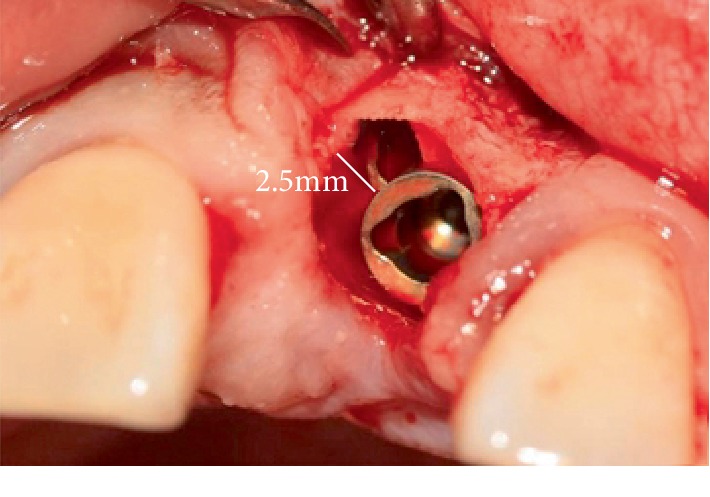
Case I: (a) reveals a 2.5 mm gap at the mesial aspect of the implant replacing the upper left incisor.
